# Genomic and phenotypic insights into ST164 *bla*_NDM-1_-positive *Acinetobacter baumannii* from intestinal colonization in China

**DOI:** 10.1186/s12866-025-03979-5

**Published:** 2025-05-07

**Authors:** Kun Wang, Weidong Zhu, Lu Gong, Xiaolu Yang, Haowei Ye, Zhenghao Lou, Jie Yang, Xiawei Jiang, Wei Li, Fangfang Tao, Hao Xu, Beiwen Zheng, Wenhong Liu

**Affiliations:** 1 School of Basic Medical Sciences, Zhejiang Chinese Medical University, Hangzhou, China; 2 Collaborative Innovation Center for Diagnosis and Treatment of Infectious Diseases, State Key Laboratory for Diagnosis and Treatment of Infectious Diseases, The First Affiliated Hospital, Zhejiang University School of Medicine, Hangzhou, China; 3 Department of Emergency Medicine, Traditional Chinese Medical Hospital of Zhuji, Zhuji, China; 4 Zhejiang Chinese Medical University School of Public Health, Hangzhou, China; 5 Jinan Microecological Biomedicine Shandong Laboratory, Jinan, China; 6 Yuhang Institute for Collaborative Innovation and Translational Research in Life Sciences and Technology, Hangzhou, China

**Keywords:** CRAB, *bla*_NDM-*1*_, Intestinal colonization, Whole-genome sequencing, Antibiotic resistance

## Abstract

**Background:**

Carbapenem-resistant *Acinetobacter baumannii* (CRAB) poses a critical global threat, especially in ICUs. Yet, reports on ST164 CRAB harboring *bla*_NDM-1_ remain scarce. This study investigates two clinical CRAB isolates, L4773hy and L4796hy, derived from intestinal colonization in Hangzhou, China, focusing on their phenotypic and genomic characteristics as well as the broader transmission of ST164 *A. baumannii*.

**Methods:**

Bacterial identification was performed using matrix-assisted laser desorption/ionization time-of-flight mass spectrometry (MALDI-TOF) mass spectrometry. Antimicrobial susceptibility was assessed via agar and broth microdilution. Whole-genome sequencing employed Illumina NovaSeq 6000 and Oxford Nanopore platforms. Resistance genes, insertion elements, transposons, and integrons were detected using ResFinder, PlasmidFinder, VFDB, ISFinder, pdifFinder, and IntegronFinder. Strains were typed by MLST, and a phylogenetic tree was constructed with kSNP3.0. Genetic environment diagrams were generated using Easyfig 2.2.5.

**Results:**

Two *bla*_NDM-1_-carrying *A. baumannii* isolates exhibiting extensive resistance to carbapenems, cephalosporins, and fluoroquinolones. Whole-genome sequencing and genetic environment analysis revealed the presence of a conserved structural sequence (IS*Aba14*-IS*Aba14*-*aphA*-IS*Aba125*-*bla*_NDM-1_-*ble*_MBL_) on their chromosomes. Phylogenetic and clonal dissemination analysis showed that ST164 CRAB is primarily distributed in China and exhibits clonal spread. Pathogenicity studies indicated that *bla*_NDM-1_-positive ST164 strains have enhanced survival under immune pressure but do not display increased virulence in infection models.

**Conclusion:**

This study provides the genomic and phenotypic characterization of intestinally colonized ST164 *bla*_NDM-1_ positive CRAB in Hangzhou, China. The elucidation of the genetic environment of *bla*_NDM-1_ further confirms the clonal dissemination of ST164 isolates, highlighting the importance of enhanced surveillance and infection control measures to mitigate the spread of these multidrug-resistant pathogens.

**Supplementary Information:**

The online version contains supplementary material available at 10.1186/s12866-025-03979-5.

## Introduction

Carbapenem-resistant *Acinetobacter baumannii* (CRAB) has emerged as one of the major pathogens responsible for hospital-acquired infections globally [1]. In recent years, carbapenem resistance in *A. baumannii* has gradually increased and is closely associated with severe clinical infections, especially in immunocompromised patients, trauma patients, and those in ICU [1]. In 2024, the World Health Organization (WHO) designated CRAB as a priority pathogen requiring urgent attention and new therapeutic strategies [2].

The carbapenem resistance in *A. baumannii* is primarily mediated by various resistance mechanisms, including the production of β-lactamases, overexpression of efflux pumps, and alterations in membrane permeability [2]. Most CRAB infections are caused by strains belonging to global clones GC1 or GC2, with GC2 accounting for most sequenced carbapenem-resistant strains [3]. The carbapenem resistance of GC2 is predominantly conferred by the *bla*_OXA-23_ β-lactamase gene, which is often located within composite transposons Tn*2006* or Tn*2009* inserted into several different chromosomal loci [4]. While *bla*_NDM-1_-positive CRAB strains have been widely reported, studies predominantly focus on clinical isolates, leaving a critical gap in understanding the role of non-clinical reservoirs, such as intestinal colonization, in the dissemination of these strains.

In this study, we report two *bla*_NDM-1_-positive CRAB isolates obtained from intestinal colonization in a patient admitted to an ICU in Hangzhou, China. Unlike previous reports focusing on clinical isolates, this study emphasizes the potential of the intestinal tract as a reservoir for *bla*_NDM-1_-positive CRAB. Genomic analysis revealed a conserved chromosomal structure harboring and highlighted the clonal dissemination of high-risk ST164 isolates within China. The findings provide valuable insights into the transmission dynamics of CRAB and underscore the importance of targeting intestinal reservoirs to mitigate the spread of multidrug-resistant pathogens.

This work systematically analyzes the antimicrobial resistance characteristics, genetic evolution, and pathogenicity of ST164 *bla*_NDM-1_-positive CRAB isolates from intestinal colonization in ICU patients in Hangzhou, China, using phenotypic and genomic approaches. It provides increasing evidence for the global dissemination of *bla*_NDM-1_-positive CRAB, offering novel perspectives on its role in hospital environments and informing future strategies for infection control and antimicrobial stewardship.

## Materials and methods

### Sample collection and bacterial culture

Two *A. baumannii* isolates, designated L4773hy and L4796hy were obtained from fecal samples of an ICU patient hospitalized at the First Affiliated Hospital of Zhejiang University in Hangzhou, China in February 2022. The patient was admitted due to severe pneumonia with complications including tracheostomy and pleural effusion. During hospitalization, the patient received antibiotic therapy with piperacillin-tazobactam for 10 days and minocycline for 7 days. Fecal samples were collected at 7-day intervals, and bacterial isolation was performed using Columbia blood agar plates, followed by incubation overnight at 37 °C under 5% CO_2_ conditions.

### Phenotype confirmation and antimicrobial susceptibility testing

The species identification of isolates L4773hy and L4796hy were confirmed using MALDI-TOF–MS (Bruker, Bremen, Germany) [5]. Antimicrobial susceptibility testing (AST) was performed using the agar dilution method and broth microdilution method. AST results were interpreted according to the 2023 standards of the Clinical and Laboratory Standards Institute (CLSI) (https://clsi.org) [6]. Breakpoints for polymyxin and tigecycline were determined based on the guidelines provided by the European Committee on Antimicrobial Susceptibility Testing (EUCAST) (https://www.eucast.org/) [7]. *Escherichia coli* ATCC 25922 and *Klebsiella pneumoniae* ATCC 700603 were used as quality control strains.

### Whole-genome sequencing (WGS)

Genomic DNA was extracted and purified using the OMEGA Bacterial DNA Kit (OMEGA Bio-tek, Norcross, GA, USA), followed by DNA sequencing on the Illumina NovaSeq 6000 (Illumina, San Diego, CA, USA) and Oxford Nanopore platforms (Oxford Nanopore Technologies, Oxford, UK) [8, 9]. Whole-genome assembly was performed using Unicycler v0.4.2 [10]. Acquired antimicrobial resistance genes (ARGs) and virulence factors were identified using ABRicate v1.0.0, based on the ResFinder, PlasmidFinder, and VFDB databases [11]. Insertion elements in the genome were analyzed using ISFinder, and transposons and integrons were examined using pdifFinder and IntegronFinder [12]. The isolates were subjected to WGS to clarify the MLST typing of the isolates based on the Pasteur MLST database [13].

### Phylogenetic and genetic environment analysis

Fasta files of all ST164 *A. baumannii* strains were downloaded from the NCBI Genome Database (as of August 2024), and ARGs and virulence factors were analyzed based on the ResFinder and VFDB databases, respectively. A phylogenetic tree was constructed using core genome single nucleotide polymorphisms (SNPs) from WGS data, with *A. baumannii* strain K09-14 as the reference. The core genome SNPs from the WGS data were used to build the phylogenetic tree using kSNP3.0. The maximum likelihood tree was visualized and modified using the online tool iTOL (https://itol.embl.de/). A SNP distance matrix was generated using snp-dists v0.7.0, with a difference of ≤ 8 indicating clonality [14].

### Pathogenicity experiment

The pathogenicity of the isolates L4773hy and L4796hy was evaluated using serum resistance assays and the *Galleria mellonella* infection model. The serum resistance assay was conducted as described previously [15], with *A. baumannii* ATCC 17978 used as the negative control. Each experiment was repeated three times. The serum resistance was measured after incubating the strains in a mixed serum from 10 healthy individuals at 37 °C for 0, 60, 120, and 180 min. For the *G. mellonella* infection model, mid-log phase cultures of L4773hy and L4796hy were adjusted to 0.5 McFarland using saline, and then diluted to a final concentration of 10^6^ CFU/mL. Each larva (10 per group) was injected with 10 µL of bacterial suspension, and survival rates were recorded every 12 h over a 7-day experimental period [16, 17]. Unpaired two-tailed Student’s t-tests were used to analyze the data, with results expressed as the mean ± standard deviation (SD).

### Accession numbers and ethical approval

The whole-genome sequences of *A. baumannii* strains L4773hy and L4796hy have been deposited in GenBank under the BioSample accession numbers SAMN43754930 and SAMN43754932. The ethical protocol for this study was approved by the Ethics Committee of the First Affiliated Hospital of Zhejiang University (Approval No: IIT20230479B).

## Results

### Species confirmation and antimicrobial susceptibility profiles

All two isolates (L4773hy and L4796hy) were confirmed as *A. baumannii* through MALDI-TOF MS. These isolates exhibited extensive resistance to carbapenems, cephalosporins, and fluoroquinolones, while remaining susceptible to tigecycline and amikacin. Polymyxin B displayed intermediate activity. MIC values are summarized in Table 1, demonstrating the multidrug-resistant profiles of these *bla*_NDM-1_-positive isolates.

**Table 1:** Antimicrobial Susceptibilities of Strains L4773hy and L4796hy

Antimicrobials	MIC Values (mg/L)	
	L4773hy	L4796hy
Imipenem	32 (R)	32 (R)
Meropenem	32 (R)	32 (R)
Ceftriaxone	> 128 (R)	> 128 (R)
Cefotaxime	> 128 (R)	> 128 (R)
Ceftazidime	> 128 (R)	> 128 (R)
Levofloxacin	8 (R)	8 (R)
Ciprofloxacin	64 (R)	64 (R)
Piperacillin-Tazobactam	> 128 (R)	> 128 (R)
Cefepime	128 (R)	128 (R)
Polymyxin B	0.5 (I)	0.5 (I)
Amikacin Sulfate	4 (S)	4 (S)
Gentamicin sulfate	2 (S)	2 (S)
Trimethoprim-Sulfamethoxazole	≤ 0.125 (S)	≤ 0.125 (S)
Tigecycline	1 (S)	1 (S)
Omadacycline	2 (S)	2 (S)

*Note*: *R* resistant, *S* susceptible, *I* intermediate

### Characterization of the genome of A. baumannii L4773hy and L4796hy

**Fig. 1:**
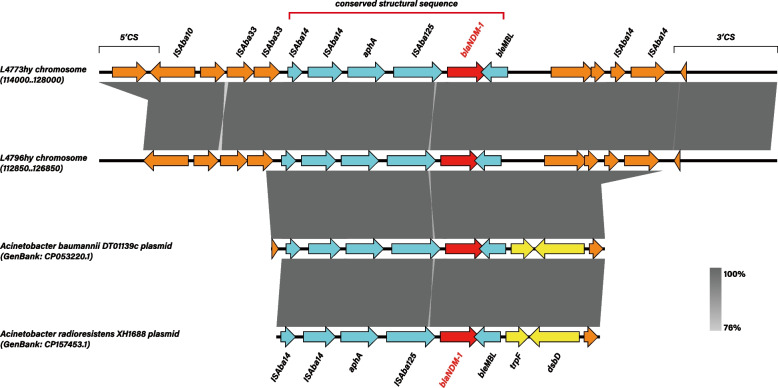
The genetic environment of bla_NDM-1_ in L4773hy and L4796hy. The conserved structural sequence (the structure within the red box.) and the genetic map of *bla*_NDM-1_ and linear comparison with *A. baumannii* strain DT01139C, *A. radioresistens* strain XH1688 based on blastn and sequence analysis. Genes are indicated as arrows. Genes, mobile elements, and other features are colored based on function classification

Whole-genome sequencing revealed that all two isolates possess a single circular chromosome and five plasmids (Table 2). Based on the MLST database, L4773hy and L4796hy were all classified as ST164. Each strain was identified by ResFinder to harbor six resistance genes, all of which are chromosomally located, as detailed in Table 3. Sequence analysis revealed a conserved structural sequence on the chromosomes of these strains, consisting of IS*Aba14*-IS*Aba14*-*aphA*-IS*Aba125*-*bla*_NDM-1_-*ble*_MBL_. NCBI BLAST analysis showed that this structure shares high genetic similarity (95% query coverage and 99% homology) with plasmid sequence fragments from *A. baumannii* strain DT01139C (Tanzania) and *A. radioresistens* strain XH1688 (China), suggesting horizontal gene transfer and chromosomal integration (Fig. 1).

**Table 2:** Genomic features of the strain L4773 hy and L4796 hy

features	L4773hy						L4796hy					
	**chromosome**	pla-1	pla-2	pla-3	pla-4	pla-5	**chromosome**	pla-1	pla-2	pla-3	pla-4	pla-5
Total number of bases (bp)	3,864,633	12,709	4,554	2,924	2,742	2,309	3,863,355	12,709	4,554	2,924	2,742	2,309
G + C content (%)	38.97	32.94	41.26	37.82	37.24	38.54	38.97	32.94	41.26	37.82	37.24	38.54
No. of protein-coding sequences	3606	14	5	5	5	1	3604	14	5	5	4	2
No. of tRNA genes	75	0	0	0	0	0	75	0	0	0	0	0

**Table 3:** Antibiotic resistance genes of strain L4773 hy and L4796 hy

Strain	Resistance Gene	Resistance Phenotype	Nucleotide Position
L4773hy	*aph(3')-VI*	Aminoglycoside resistance	119,136–119,915
	*bla*_NDM-1_	Carbapenem resistance	121,193–122,005
	*bla*_ADC-25_	Cephalosporin resistance	298,458–299,624/1,195,938–1,197,089
	*bla*_OXA-23_	Carbapenem resistance	729,184–730,005/1,262,024–1,262,845
	*bla*_CARB-16_	Beta-lactam resistance	1,894,809–1,895,705
	*bla*_OXA-91_	Carbapenem resistance	2,188,461–2,189,285
L4796hy	*aph(3')-VI*	Aminoglycoside resistance	117,851–118,630
	*bla*_NDM-1_	Carbapenem resistance	119,908–120,720
	*bla*_ADC-25_	Cephalosporin resistance	297,173–298,339/1,194,653–1,195,804
	*bla*_OXA-23_	Carbapenem resistance	727,899–728,720/1,260,739–1,261,560
	*bla*_CARB-16_	Beta-lactam resistance	1,893,524–1,894,420
	*bla*_OXA-91_	Carbapenem resistance	2,187,176–2,188,000

### Phylogenetic and clonal dissemination analysis

**Fig. 2:**
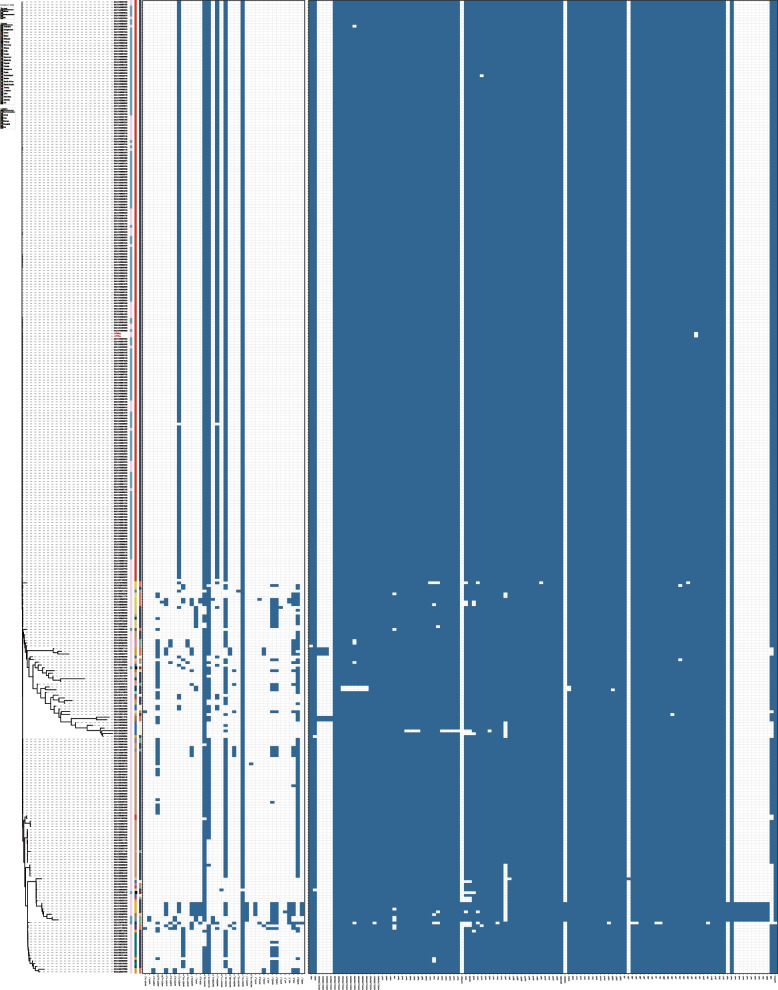
The maximum likelihood core gene phylogenetic tree of ST164 *A. baumannii*. The maximum likelihood core-gene phylogenetic tree of ST164 *A. baumannii*, generated by kSNP3.0 using *A. baumannii* K09-14 as a reference genome. The sources of strains are identified as clinical, environment, and not available and are marked with pink, blue, and green squares, respectively. The heatmap displays the types of antibiotic resistance genes and virulence genes in each strain. Blue indicates that the isolate carries such genes and colorless means that the genes are not present

**Fig. 3:**
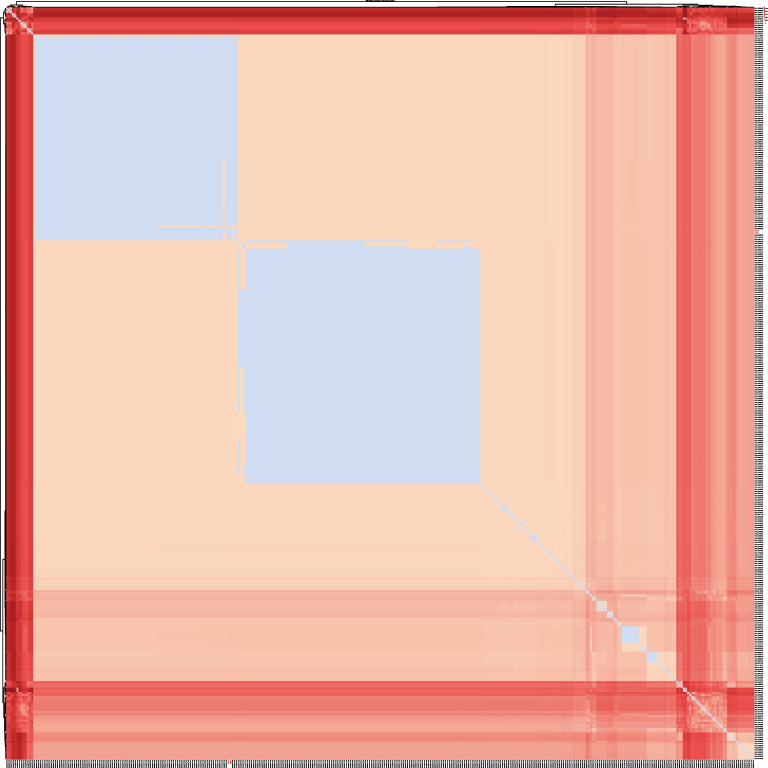
The SNP distance matrix of ST164 *A. baumannii*. The SNP distance matrix features light blue segments, signifying SNP differences that are less than or equal to 8. Based on SNP analysis, *A. baumannii* strains with an SNP difference of less than or equal to 8 are considered to belong to the same clone

Phylogenetic analysis of 353 publicly available ST164 *A. baumannii* genomes revealed that 218 strains were *bla*_NDM-1_-positive, with a positivity rate of 61.75%. Among these, isolates from China formed a distinct clade, with most carrying *bla*_NDM-1_ (Fig. 2). The maximum likelihood phylogenetic tree showed that the Hangzhou isolates cluster closely with other Chinese strains, suggesting clonal dissemination. SNP distance matrix analysis confirmed high genetic relatedness among Chinese ST164 isolates, with a majority exhibiting ≤ 8 SNP differences, indicative of clonality (Fig. 3). Geographic distribution analysis highlighted the predominance of ST164 *bla*_NDM-1_-positive isolates in Hangzhou, accounting for 95.37% of such strains in China.

The results indicated that ST164 *A. baumannii* is predominantly distributed in Asia, with the highest prevalence in China, followed by Southeast Asian countries and India, and a minority in Africa, North America, Europe, and Oceania. The strains isolated in China clustered together, most of which were from clinical samples and hospital environments. These strains primarily harbored six resistance genes: *aph(3')-VI*, *bla*_NDM-1_, *bla*_ADC-25_, *bla*_OXA-23_, *bla*_CARB-16_, and *bla*_OXA-91_. Strains from other regions were mostly isolated from clinical samples and exhibited almost no *bla*_NDM-1_ carriage. The virulence factors of ST164 *A. baumannii* from China were found to be almost identical to those from other regions. Genetic environment analysis of the *bla*_NDM-1_ fragment in all *bla*_NDM-1_-carrying ST164 *A. baumannii* strains from China revealed high genetic similarity (Fig. S1). Further SNP distance matrix analysis of the core genome indicated that the strains from China were closely related, with the predominant strains being derived from two main clones.

### Pathogenicity experiment results

**Fig. 4:**
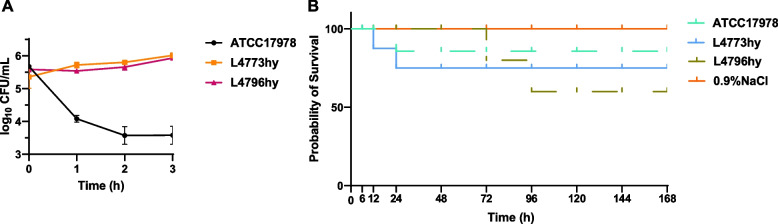
Pathogenicity characterization and time-kill curves of *A. baumannii* strains. **A** Serum resistance assay of *A. baumannii* strains L4773hy and L4796hy. **B** Survival curve of the larval infection model of *Galleria mellonella*

All isolates demonstrated high serum resistance, maintaining viability after 180 min of incubation in pooled human serum (Fig. 4A). However, in the *G. mellonella* infection model, no significant difference in virulence were observed when compared to the standard *A. baumannii* strain ATCC 17978 (Fig. 4B). These findings suggest that *bla*_NDM-1_-positive ST164 strains may exhibit higher survival rates under host immune pressure, but further studies are needed to explore their association with clinical outcomes. Additionally, they do not show increased virulence in the *G. mellonella* infection model.

## Discussion

Carbapenem-resistant *Acinetobacter baumannii* remains one of the most formidable challenges in global healthcare, particularly in ICU settings. Since the first report of *bla*_NDM-1_-carrying multidrug-resistant *A. baumannii* in India in 2010 [18], there have been increasing reports of the global spread of such strains [2]. Due to their ability to readily adapt to hospital environments, particularly in ICUs, where they can easily survive and rapidly spread between patients and the environment [19], this species has become one of the major pathogens responsible for healthcare-associated infections. In this study, we report two ST164 CRAB strains from intestinal colonization, highlighting the potential of the gastrointestinal tract as a reservoir for multidrug-resistant pathogens and a critical factor in nosocomial dissemination [20].

### Genetic mechanisms

Previous reports have indicated that resistance to carbapenems in *A. baumannii* is typically mediated by *bla*_OXA-23_, *bla*_OXA-24_, and *bla*_OXA-58_ [21]. In China, *bla*_OXA-23_ has historically been the main factor responsible for carbapenem resistance in *A. baumannii* [22, 23], and co-carriage of *bla*_OXA-23_ and *bla*_NDM-1_ is relatively rare [24], especially when both are integrated into the chromosome rather than located on plasmids. Moreover, the *bla*_NDM-1_ gene is usually found in the Tn*125* transposon [25], but in this study, no integrons or classical Tn*125* transposon structures were found. Subsequent sequence analysis revealed that all strains contained a conserved structural sequence on their chromosomes, which shares high genetic similarity with plasmid sequence fragments carrying *bla*_NDM-1_ from various sources of *Acinetobacter* sp. (Fig. 1). Notably, the genes carried by these plasmid sequences exhibit significant differences compared to those carried on the chromosomes of strains like L4773hy. Specifically, the *trpF* and *dsbD* genes located downstream of *ble*_MBL_ in the plasmid sequences are absent from the chromosomes of these two strains.This observation suggests that the *bla*_NDM-1_ gene in the strains reported in this study likely originated from plasmids of other *Acinetobacter* sp. carrying *bla*_NDM-1_, which were integrated into the chromosome. However, during the integration process, point mutations occurred, resulting in the loss of the *trpF* and *dsbD* genes from the originally conserved structural sequence on the plasmid. This mechanism differs from the previously reported transmission of *bla*_NDM-1_ in *A. baumannii* via the *Tn125* transposon [26]. The conserved sequence identified in this study was also observed in the Tn*125*-like structures reported by Fu et al., who hypothesized that the formation and transmission of this structure are mediated by the insertion of two *ISAba14* elements flanking the *bla*_NDM-1_ gene into the bacterial chromosome [27]. The conserved chromosomal structure (IS*Aba14*-IS*Aba14*-*aphA*-IS*Aba125*-*bla*_NDM-1_-*ble*_MBL_) identified in this study differs from the typical Tn*125*-associated *bla*_NDM-1_ structures reported globally. This suggests an alternative genetic mechanism for the dissemination of *bla*_NDM-1_ in ST164 isolates, possibly through chromosomal integration mediated by insertion sequences such as IS*Aba14*.Therefore, the mechanism by which this structure mediates the transmission of *bla*_NDM-1_ in these strains requires further investigation.

### Transmission and clinical relevance

Most *bla*_NDM-1_-positive CRAB isolates previously reported from Hangzhou and other regions of China were derived from clinical infections, such as bloodstream and respiratory tract infections, with fewer reports of isolates from fecal or rectal swabs. In contrast, the isolates in this study were recovered from fecal samples, supplementing novel insights into the role of intestinal colonization in the epidemiology of CRAB. Recent reports have described a new international clone, referred to as IC11 (ST164^Pas^/ST1418^Ox^) [28], which has been identified in Kenya [29], Thailand [30], Egypt [31], and China [24]. This aligns with the geographical distribution of ST164 CRAB presented in this study. However, it is noteworthy that most ST164 strains from China carry *bla*_NDM-1_, and they are genetically very closely related, a phenomenon not observed in ST164 strains from other regions. The SNP matrix analysis revealed that the majority of *bla*_NDM-1_-carrying ST164 isolates from China originated from two main clones, suggesting clonal transmission. Further analysis found that 213 *bla*_NDM-1_-carrying ST164 *A. baumannii* strains are all distributed in Hangzhou, Zhejiang, accounting for 95.37% of all ST164 strains in China. Almost all ST164 strains were sourced from clinical samples or hospital environments, demonstrating a high degree of clonality. This supports the hypothesis that *bla*_NDM-1_-positive ST164 strains are undergoing local clonal expansion, likely driven by selective pressure from widespread antibiotic use in healthcare settings. Furthermore, geographic distribution analysis confirmed that Hangzhou is a hotspot for *bla*_NDM-1_-positive ST164 isolates, accounting for the majority of such strains in China. Hangzhou, as a large city with abundant medical resources and high patient mobility, has facilitated the spread of drug-resistant bacteria [32].These findings align with recent reports of ST164 emerging as a high-risk lineage in Asia.

In the investigation of the pathogenicity of these resistant strains, it was found that they exhibited high resistance to serum killing. This suggests that these strains possess strong survival capabilities when confronted with host immune responses and antibiotic pressure, presenting challenges in clinical treatment. However, in the *G. mellonella* infection model, these resistant strains did not show significantly higher virulence compared to the standard *A. baumannii* strain ATCC 17978, indicating no enhanced pathogenicity. This finding suggests that, although these strains have a significant advantage in antibiotic resistance, their pathogenic potential may not be correspondingly increased. This highlights that *bla*_NDM-1_-positive *A. baumannii* strains exhibiting high serum resistance are capable of surviving in antibiotic-intensive hospital environments while enhancing their persistence within the host (such as humans). This enables them to remain difficult to clear by the immune system during antibiotic treatment, potentially leading to chronic or recurrent infections, thus increasing their transmission risk within hospital settings. While the isolates in this study did not demonstrate significantly enhanced virulence in infection models, this does not imply a lack of transmissibility, they could still be spread by asymptomatic carriers.The intestinal colonization of *bla*_NDM-1_-positive ST164 *A. baumannii* poses a significant challenge for infection control. Colonized patients can serve as reservoirs for horizontal gene transfer and nosocomial transmission, particularly in high-risk environments such as ICUs. The high serum resistance observed in these isolates underscores their ability to evade host immune responses, enhancing their persistence in both patients and hospital environments. It is recommended that ICUs conduct stool screening for *bla*_NDM-1_ in high-risk patients, along with environmental disinfection and contact isolation measures, to prevent the spread of intestinal colonizing bacteria. Although the *Galleria mellonella* infection model did not indicate increased virulence compared to standard strains, the survival advantage conferred by *bla*_NDM-1_ and other resistance genes highlights the importance of robust antimicrobial stewardship and targeted surveillance to mitigate their spread.

This study does have some limitations, including the need for further exploration of the transmission mechanisms of *bla*_NDM-1_ in non-transposon structures on the chromosome. Additionally, when conducting the genetic environment analysis of *bla*_NDM-1_-carrying ST164 *A. baumannii* in China, the samples included incomplete gene fragments, which may not fully represent the spread of *bla*_NDM-1_ and its conserved structure in China. Moreover, the use of the *G. mellonella* infection model may not completely simulate the complexity of human infections. Establishing a murine infection model could provide further insights into the virulence levels and pathogenesis of these strains.

## Conclusion

In conclusion, we have reported two ST164 CRAB isolates L4773hy and L4796hy, which were sourced from intestinal colonization. The findings emphasize the critical role of the gastrointestinal tract as a reservoir for CRAB and highlight the urgent need for enhanced infection control measures and antimicrobial stewardship to curb the spread of these multidrug-resistant pathogens.

## Supplementary Information

Supplementary Material 1: Figure S1 Genetic environment analysis of the bla_NDM-1_ fragment in all ST164 *A. baumannii* isolates from China. Genes are indicated as arrows. Genes, mobile elements, and other features are colored based on function classificati.

Supplementary Material 2.

## Data Availability

The whole-genome sequences of A. baumannii strains L4773hy and L4796hy have been deposited in GenBank under the BioSample accession numbers SAMN43754930 and SAMN43754932.

## Abbreviations

CRAB: Carbapenem-resistant *Acinetobacter **baumannii*
ICU: Intensive Care Unit
WHO: World Health Organization
MALDI-TOF–MS: Matrix-assisted laser desorption/ionization time-of-flight mass spectrometry
CLSI: Clinical and Laboratory Standards Institute
EUCAST: European Committee on Antimicrobial Susceptibility Testing
WGS: Whole-genome sequencing
ARGs: Antimicrobial resistance genes
SNPs: Single nucleotide polymorphisms
SD: Standard deviation
