# Supplementary material for: Genomic and phenotypic insights into ST164 blaNDM-1-positive Acinetobacter baumannii from intestinal colonization in China
Source: BMC Microbiol. 2025 May 7;25:272. doi: 10.1186/s12866-025-03979-5 (PMC12057158; doi:10.1186/s12866-025-03979-5)
Supplement: Supplementary file 1 — Supplementary Material 1: Figure S1 Genetic environment analysis of the blaNDM-1 fragment in all ST164 A. baumannii isolates from China. Genes are indicated as arrows. Genes, mobile elements, and other features are colored based on function classificati. [file 12866_2025_3979_MOESM1_ESM.pdf]

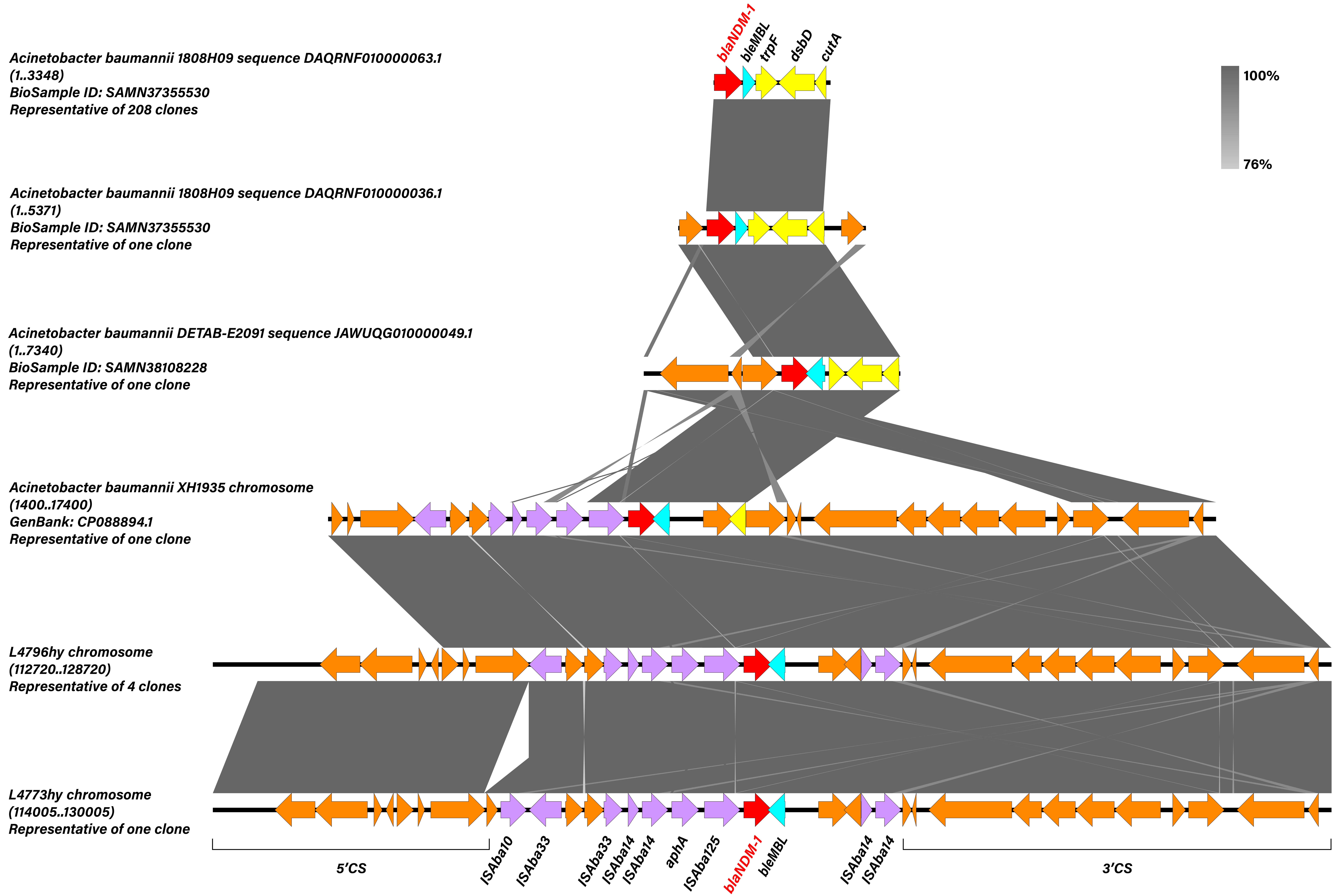
Acinetobacter baumannii 1808H09 sequence DAQRNF010000063.1
(1..3348)
BioSample ID: SAMN37355530
Representative of 208 clones
Acinetobacter baumannii 1808H09 sequence DAQRNF010000036.1
(1..5371)
BioSample ID: SAMN37355530
Representative of one clone
Acinetobacter baumannii DETAB-E2091 sequence JAWUQG010000049.1
(1..7340)
BioSample ID: SAMN38108228
Representative of one clone
Acinetobacter baumannii XH1935 chromosome
(1400..17400)
GenBank: CP088894.1
Representative of one clone
L4796hy chromosome
(112720..128720)
Representative of 4 clones
L4773hy chromosome
(114005..130005)
Representative of one clone
blaNDM-1
bleMBL
trpF
dsbD
cutA
100%
76%
5′CS
ISAba10
ISAba33
ISAba33
ISAba14
ISAba14
aphA
ISAba125
blaNDM-1
bleMBL
ISAba14
ISAba14
3′CS
